# NF-κB Affects Proliferation and Invasiveness of Breast Cancer Cells by Regulating CD44 Expression

**DOI:** 10.1371/journal.pone.0106966

**Published:** 2014-09-03

**Authors:** Shannon M. Smith, Yi Lisa Lyu, Li Cai

**Affiliations:** 1 Cell and Developmental Biology Graduate Program, Rutgers University, Piscataway, New Jersey, United States of America; 2 Department of Pharmacology, Robert Wood Johnson Medical School, Rutgers University, Piscataway, New Jersey, United States of America; 3 Department of Biomedical Engineering, Rutgers University, Piscataway, New Jersey, United States of America; Stony Brook University, United States of America

## Abstract

NF-κB plays an important role in cancer initiation and progression. CD44, a cell surface glycoprotein, is involved in many cellular processes including cell adhesion, migration and proliferation. However, whether and how the two molecules interact in breast cancer is not clear. In recent years, the up-regulation of CD44 has served as a marker for tumor initiating cells in breast cancer and other cancer types. Despite the important role of CD44 in cellular processes and cancer, the mechanism underlying CD44 up-regulation in cancers remains poorly understood. Previously, we have identified a novel *cis*-element, CR1, located upstream of the CD44 promoter. We demonstrated that NF-κB and AP-1 are key *trans*-acting factors that interact with CR1. Here, we further analyzed the role of NF-κB in regulating CD44 expression in triple negative breast cancer cells, MDA-MB-231 and SUM159. Inhibition of NF-κB by Bay-11-7082 resulted in a reduction in CD44 expression. CD44 repression via NF-κB inhibition consequently decreased proliferation and invasiveness of breast cancer cells. These findings provide not only new insight into the molecular mechanism underlying CD44 regulation but also potential therapeutic targets that may help eliminate chemo- and radiation-resistant cancer cells.

## Introduction

Breast cancers are known to contain a heterogeneous population of cells. Within a tumor, there is a small subset of cells with a unique cell surface marker signature (e.g., up-regulation of CD44 and down-regulation of CD24) as well as characteristics similar to stem cells such as the ability to self-renew, differentiate and they have been shown to be chemo-and radiation resistant [1]–[6]. These cells, known as cancer stem-like cells or tumor initiating cells (TICs), have been observed in other cancers including prostate, pancreatic, brain and leukemia [7]–[9], making CD44 an important target for cancer therapies.

CD44 is a cell surface glycoprotein that is ubiquitously expressed on most cells throughout the body [5], [6]. CD44 is involved in cellular processes including cell-cell and cell-extracellular matrix adhesion, migration, proliferation, differentiation and survival [5], [6], [10], [11]. Studies have shown that human acute myeloid leukemic stem cells can be eradicated by targeting CD44 [9]. In addition, CD44 repression by miR-34a inhibits prostate TICs and metastasis [12].

Despite intense research focused on CD44 as a target for cancer therapies, the mechanism by which the protein is up-regulated in cancer and TICs is not well understood. In our recently published study, we have identified an evolutionarily conserved region (CR1) located upstream of the CD44 transcription start site, that functions as a *cis-*element [13]. We have demonstrated that CR1 has the ability to direct reporter gene expression in a cell-specific manner. We showed that CR1 activity is modulated by the transcription factors NF-κB and AP-1 via electrophoretic mobility shift assays (EMSA), EMSA supershift, and chromatin immunoprecipitation (ChIP) assays. Site directed mutagenesis of the NF-κB and AP-1 binding sites diminished the ability of CR1 to direct reporter gene expression in breast cancer cells [13].

The NF-κB family (RelA (p65), c-Rel, RelB, p50/105 and p52/100) has been at the forefront of cancer research [14], [15]. There are more than 100 known targets of NF-κB, including CD44 [16]. NF-κB exists as a homo- or heterodimer in the cytoplasm, inhibited by bound IκB proteins. It is not until IκB is phosphorylated that NF-κB can enter the nucleus, bind to DNA and activate transcription of its target genes [14], [17], [18]. Recent studies have demonstrated that CD44 expression and NF-κB activation correlate with poor radiation response and shorter survival in glioblastoma patients [19]. However, the mechanism underlying CD44 regulation by NF-κB is not clear.

In this study, we examine the effect of NF-κB inhibition on CD44 expression and the activities associated with CD44 dysregulation, including cell proliferation and invasiveness in breast cancer cells. We show that in triple negative breast cancer cells (e.g., MDA-MB-231 and SUM159 cells), inhibition of NF-κB via the chemical compound Bay-11-7082 results in CD44 repression. Furthermore, NF-κB inhibition and subsequent CD44 repression decreases cell proliferation and invasiveness of breast cancer cells. Thus, our findings provide new insights into the mechanism underlying CD44 up-regulation in breast cancers and potential therapeutic strategy against breast cancers.

## Materials and Methods

### Cell Lines

The breast cancer cell lines SUM159 and MDA-MB-231 were describe previously [8]. SUM159 cells (Asterand Inc. Detroit, MI) and MDA-MB-231 cells (ATCC) were cultured according to the guidelines from the suppliers. All cell lines were maintained at 37°C in a humidified incubator with 5% CO_2_.

### Bay-11-7082 Treatment

Bay-11-7082 (Calbiochem) in DMSO was diluted in serum free medium to a concentration of 1.0 mM. As a control, 10 µL of DMSO was added per 1.0 ml of media. This was the maximum amount of DMSO cells were exposed to for Bay-11-7082 treatment.

### Electrophoretic mobility shift assay (EMSA)

A double stranded DNA probe with the sequence 5′–GATCCGGCAGGGGAATCTCCCTCTC-3′ was labeled with the 3′ Biotin End Labeling Kit (Thermo Scientific) as per manufacturer's suggestions. Nuclear extracts were collected from each breast cancer cell line using NE-PER nuclear and cytoplasmic extraction reagents (Thermo Scientific). Binding reactions were performed using 5 µg of nuclear extract from cells and detected using the LightShift Chemiluminescent EMSA kit (Thermo Scientific) per manufacturer's recommendations. DNA-protein complexes were run on 6% non-denaturing poly-acrylamide gels and transferred onto Biodyne Plus membrane (Pall). Membranes were cross-linked in a UV imager for 15 minutes.

### Western Blot

Western blots were performed using 15 µg cytoplasmic extract. Cytoplasmic extracts were collected using NE-PER (Thermo Scientific). Cytoplasmic extracts in SDS-PAGE sample buffer, were incubated at 95°C for 5 min. Samples were run on a 10% SDS-PAGE gel and transferred onto nitrocellulose. Membranes were incubated in 5% non-fat dry milk for 1 hr and incubated with primary antibody (CD44 (Santa Cruz) or alpha-Tubulin (DSHB) over night at 4°C. Membranes were incubated with secondary antibody (Santa Cruz) for 1 hr at room temperature. Membranes were exposed with a chemiluminescence kit (Thermo Scientific) and imaged.

### qRT-PCR

RNA was isolated from cells using Tri-Reagent (Ambion). cDNA was prepared by reverse transcription using the qScript cDNA SuperMix (Quanta), and used as a template for RT-PCR (SYBR Green FastMix (Applied Biosystems)). RT-PCR reaction was run on a Roche 480 96 well LightCycler using primer sequences obtained from the Harvard Primer Bank (**Table 1**). Threshold cycles were normalized relative to GAPDH expression. Experiments are the mean of 2 independent experiments done in triplicate. Error bars represent the standard deviation of the mean.

**Table 1:** qPCR primer sequences obtained from Harvard Primer Bank (http://pga.mgh.harvard.edu/primerbank/).

Name	Primer	Sequence
NF-κB2	Forward	ATGGAGAGTTGCTACAACCCA
	Reverse	CTGTTCCACGATCACCAGGTA
CD44	Forward	TGCCGCTTTGCAGGTGTATT
	Reverse	CCGATGCTCAGAGCTTTCTCC
BCL-XL	Forward	GATCCCCATGGCAGCAGTAAAGCAAG
	Reverse	CCCCATCCCGGAAGAGTTCATTCACT
cMyc	Forward	ATGGCCCATTACAAAGCCG
	Reverse	TTTCTGGAGTAGCAGCTCCTAA

### Immunocytochemistry

For immunocytochemistry, cells were plated on PLL treated coverslips and incubated for 24 hours and then fixed to coverslips using 4% paraformaldehyde, blocked with 10% Donkey Serum (Jackson Immunology) and then incubated with the primary antibody for 2 hours at room temperature. The following antibodies were used CD44 (Chemicon); Ki67 (BD Pharmingen). Following incubation with primary antibody, cells were incubated with a fluorescent secondary antibody (Jackson Immunology) for 60 minutes at room temperature. Nuclei were stained with Hoechst33342. Cell counts were obtained from independent experiments performed in triplicate. Error bars represent the standard deviation of the mean.

### Measurement of Cell Size

Cells were measured using ImageJ measurement tool. Images of cells were taken on Zeiss AxioImager A1 fluorescence microscope. Only cells that could be completely identified and were not blocked by other cells or cut off by the image were measured. Measurements were taken from the furthest two points on the cell. A minimum of 200 cells were measured from 2 independent experiments. Error bars represent the standard deviation of the mean.

### Cell Proliferation Assay

Cell proliferation assay was performed using CyQuant Cell Proliferation kit (Life Technologies) as per manufacturer's recommendation. Cells were seeded in 96 well plates at different densities and left for 24 hrs in 37°C incubator. Cells were treated with DMSO or Bay-11-7082 and incubated for 24, 48 or 72 hrs. Assay was read on a Tecan Infinite M200 Pro 96 well plate reader. Data was compared to standard curve. Results of each data time point represent the mean of 3 independent experiments. A standard curve was created for each cell type. Cell number was calculated from the standard curve. Fold change was calculated by the following equation:
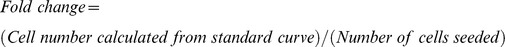



### Invasion Assay

Invasion assays were performed as per manufacturer's recommendations (BD Biosiences). MDA-MB-231 cells and SUM159 cells were treated with 2.5 µM Bay-11-7082 for 72 hrs and 48 hrs respectively. Cells were detached with trypsin, counted and resuspended in serum free media at a concentration of 50,000 cells/ml. Complete media was placed in wells as chemo-attractant and 0.5ml of resuspended cells were seeded into control chambers and BD BioCoat Matrigel invasion chambers and incubated for 24 hrs. Following incubation, media was removed from the wells and chambers, cells were fixed in 90% methanol for 3 min. Cells were stained with Hoechst33342. Membranes were removed, adhered to slides, and then imaged. Cells were counted and percent *migration* and *invasion* was calculated the following equations:
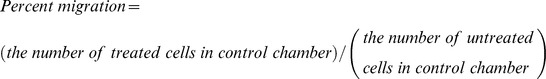


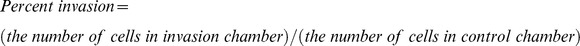



### Data Quantification

Results of each data time points were from at least 3 samples. Error bars represent the standard error of the mean. In cases where results were tested for statistical significance, a student's t-test was applied.

## Results

### Chemical compound Bay-11-7082 inhibits NF-κB binding to DNA in breast cancer cells

To determine the role of NF-κB in regulating CD44 expression, NF-κB activation was inhibited using the chemical compound Bay-11-7082. Bay-11-7082 has previously been shown to inhibit NF-κB binding to DNA by preventing phosphorylation of the Inhibitor of κB (IκB) by the IκB Kinase (IKK) [20]–[23]. Inhibiting phosphorylation of IκB inhibits the activation of NF-κB and subsequent binding to DNA. We chose breast cancer cells MDA-MB-231 and SUM159 for this study as both are triple negative breast cancer cells (ER^-^, PR^-^, HER2^-^) with high levels of CD44 expression and contain a subpopulation of cells characterized as TICs [24], [25].

Breast cancer cells were treated with Bay-11-7082 at various concentrations for 24, 48 or 72 hrs to determine which concentration and duration of treatment have the greatest effect on inhibiting NF-κB activation. Treatment with DMSO was used as a control. Electrophoretic mobility shift assays (EMSA) were performed to determine the ability of NF-κB to bind to DNA following treatment. A double stranded, biotin labeled oligonucleotide corresponding to the NF-κB binding site was used to assess binding activity.

**Figure 1:**
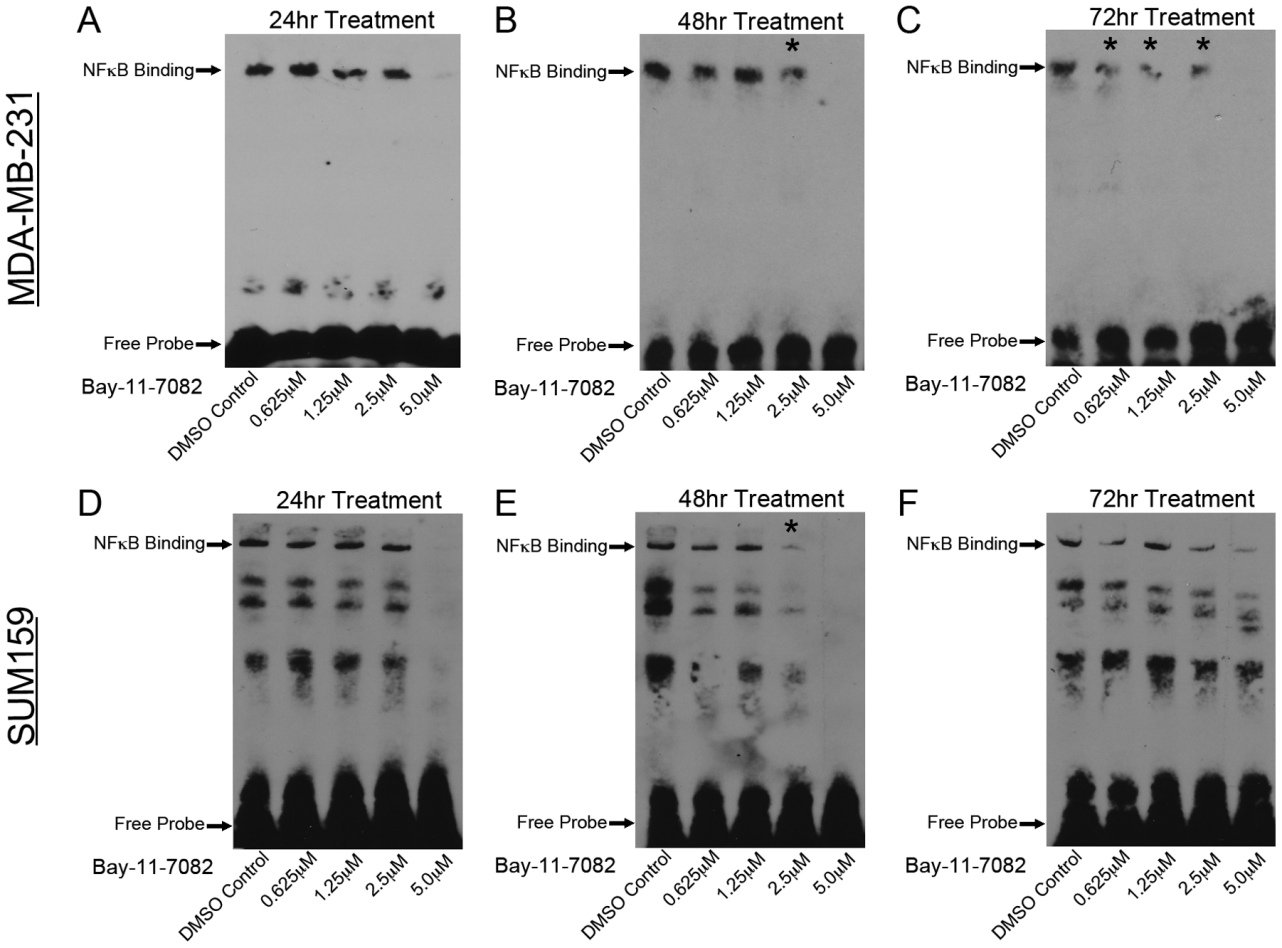
Bay-11-7082 inhibits NF-κB binding to DNA in breast cancer cells. Breast cancer cells, MDA-MB-231 (**A–C**) and SUM159 (**D–F**), were treated with either a DMSO control or Bay-11-7082 at different concentrations (0.625 µM - 5.0 µM) for 24, 48 or 72 hrs. Electrophoretic mobility shift assays (EMSA) showed decreased NF-κB binding at 2.5 µM of Bay-11-7082 treatment for 72 hrs in MDA-MB-231 cells (indicated by an asterisk in **C**) and 48 hrs treatment in SUM159 cells (indicated by an asterisk in **E**). NF-κB binding was completed abolished at 5.0 µM concentration, except 72 hrs treatment in SUM159 cells (**F**).

In MDA-MB-231 cells, treatment with 5.0 µM Bay-11-7082 resulted in a diminished band at all three time points (**Fig. 1A–C**), indicating an inhibition effect of NF-κB binding. A strong band in EMSA was seen in 24 and 48 hrs of treatment with DMSO control and Bay-11-7082 at 0.625 µM and 1.25 µM (**Fig. 1A–C**), suggesting that DMSO control and low concentrations of Bay-11-7082 have no obvious effect on NF-κB binding. Noticeable decrease in EMSA bands was observed at 2.5 µM Bay-11-7082 after 48 hrs (indicated by asterisks in **Fig. 1B**); and after 72 hrs treatment decreased NF-κB binding was seen at all concentrations (indicated by asterisks in **Fig. 1C**).

In SUM159 cells, loss of NF-κB binding was observed with 5.0 µM Bay-11-7082 treatment after 24 and 48 hrs (**Fig. 1D,E**), with little change in binding occurring at 0.625 µM and 1.25 µM concentration. A significant decrease in NF-κB binding was observed with 2.5 µM treatment after 48 hrs (**Fig. 1E**). Interestingly, weak EMSA bands could be seen with 2.5 µM and 5.0 µM Bay-11-7082 after 72 hrs of treatment (**Fig. 1F**), suggesting that SUM159 cells may have developed a drug resistance to Bay-11-7082 after 72 hrs of treatment.

Although applying higher concentrations of Bay-11-7082 (e.g., 5.0 and 10.0 µM) showed the greatest effect on NF-κB binding at all-time points, a live/dead cell assay showed toxicity of the treatment, which resulted in significant levels of cell death in both cell types (**Fig. S1**). Based on these observations, the maximum concentration of Bay-11-7082 used in further analyses was determined at 2.5 µM.

### NF-κB inhibition results in CD44 repression

**Figure 2:**
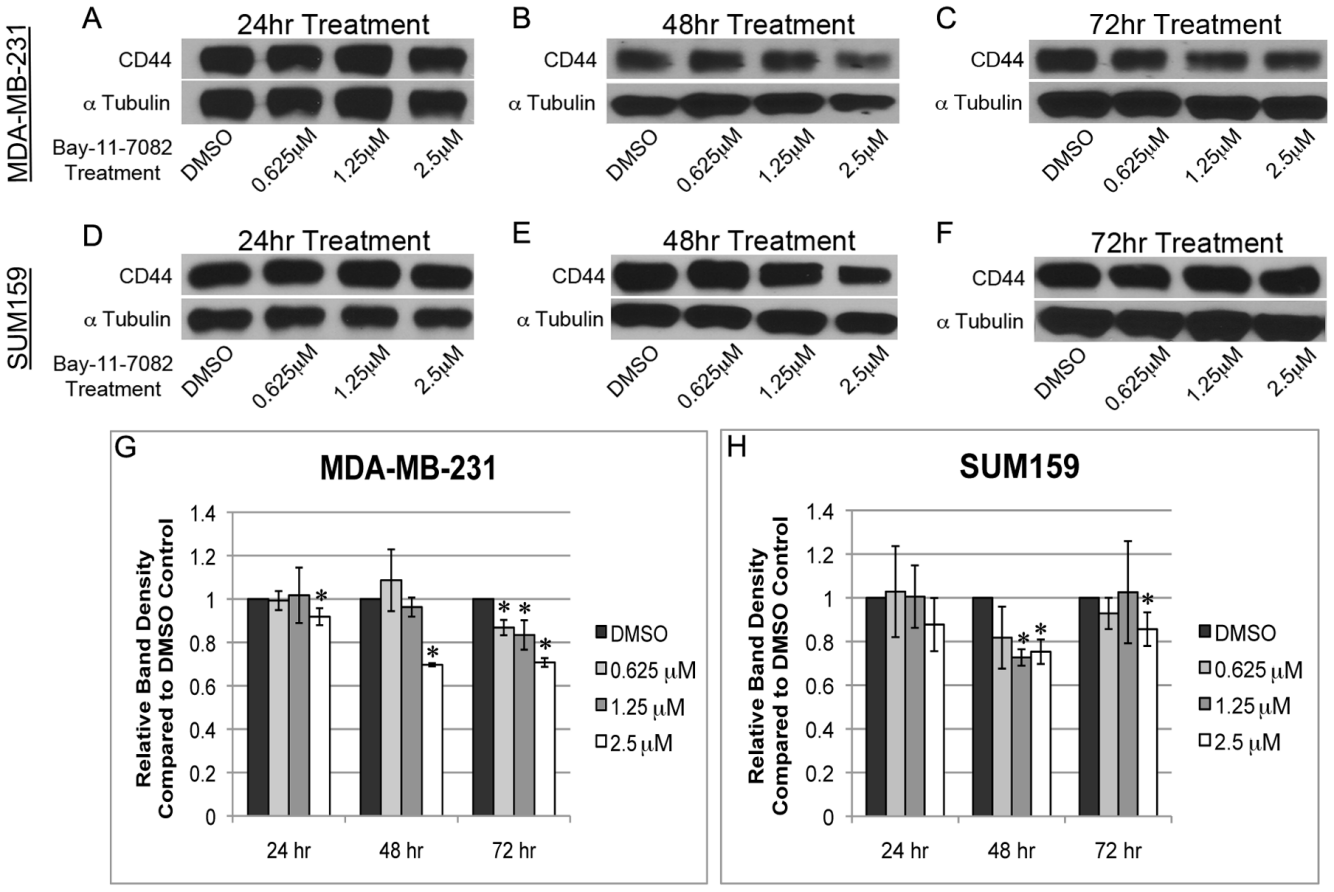
Bay-11-7082 treatment leads to NF-κB inhibition and down-regulation of CD44 in breast cancer cells. Western blots showed that inhibition of NF-κB decreases CD44 expression in MDA-MB-231 (**A–C**) and SUM159 cells (**D–F**). Quantification showed a significant decrease in CD44 protein expression in MDA-MB-231 (**G**) and SUM159 cells (**H**). Band density was quantified using ImageJ (n = 3; * p ≤ 0.01).

Next, we assessed the effect of NF-κB inhibition on CD44 expression by Western blotting using the cytoplasmic extracts of Bay-11-7082 treated cells at each of the three time points individually. Resulting bands were analyzed using ImageJ to quantify the relative amount of CD44 protein compared to the control DMSO treatment. In MDA-MB-231 cells, CD44 expression decreased 10% after 24 hrs treatment at 2.5 µM while lower concentrations (0.625 µM and 1.25 µM) did not show a noticeable difference (**Fig. 2A,G**). CD44 expression decreased ∼30% after 48 hrs treatment at 2.5 µM, (**Fig. 2B,G**). A significant decrease in CD44 expression was observed at all concentrations after 72 hrs with the greatest reduction of CD44 expression (∼30%) occurring at 2.5 µM treatment (**Fig. 2C,G**). In SUM159 cells, no changes in CD44 expression were seen following 24 hrs of treatment (**Fig. 2D,H**). A significant decrease in CD44 expression (∼28% and 25%) was detected after 48 hrs treatment at 1.25 µM and 2.5 µM, respectively (**Fig. 2E,H**). Interestingly, after 72 hrs, a decrease in CD44 expression was only seen with 2.5 µM Bay-11-7082 treatment (**Fig. 2F,H**). This result may suggest that CD44 expression recovers after SUM159 cells develop a drug resistance to Bay-11-7082 after 72 hrs of treatment.

**Figure 3:**
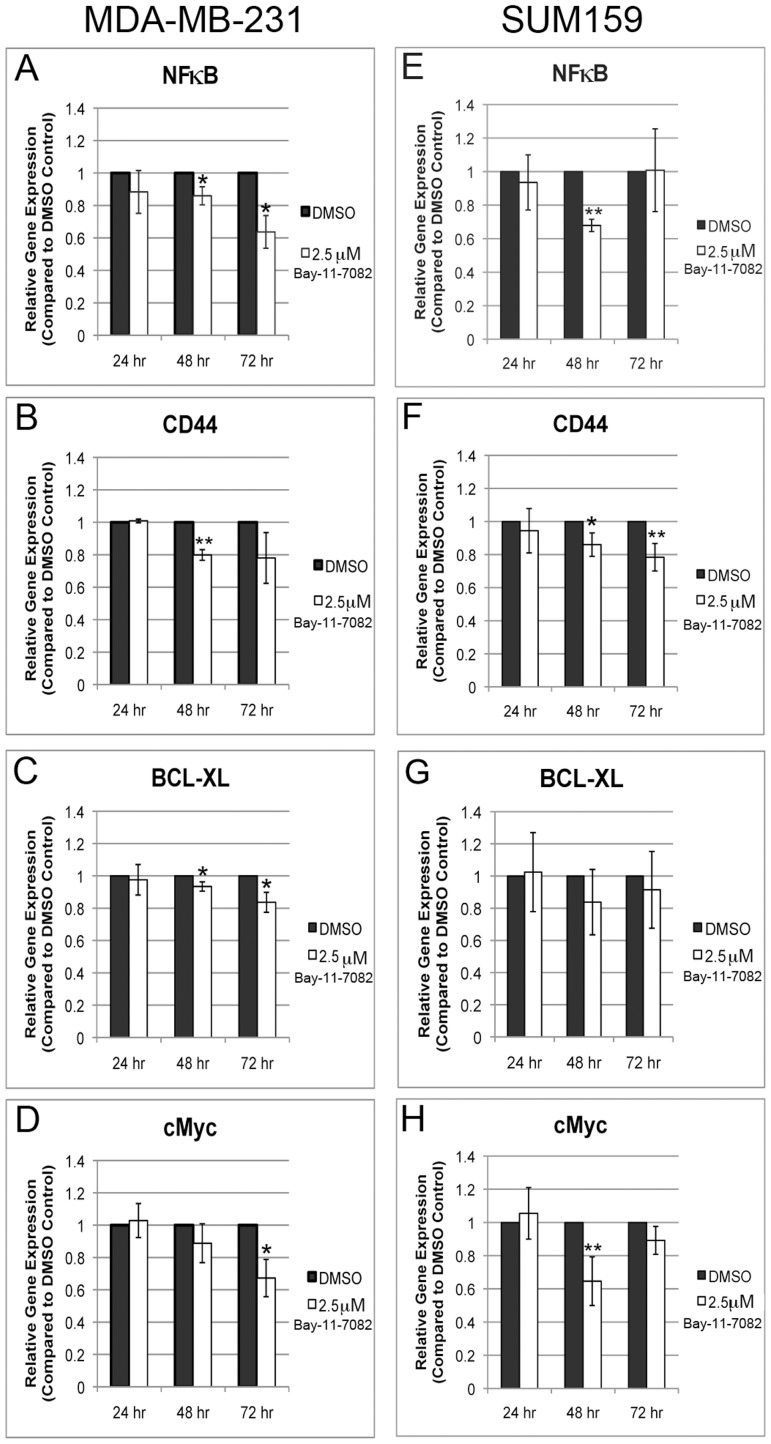
Bay-11-7082 treatment decreases RNA expression of NF-κB and CD44 in breast cancer cells. Real-time PCR (qPCR) analyses showed Bay-11-7082 treatment decreases the expression of NF-κB and its target genes (e.g., CD44, BCL-XL, and cMyc) in MDA-MB-231 (**A–D**) and SUM159 cells (**E–H**) (n = 3; * p≤0.05, ** p≤0.01).

To further confirm the effects of Bay-11-7082 on NF-κB inhibition, the mRNA level of NF-κB and its known key targets, e.g., CD44, BCL-XL, and cMyc, was determined using quantitative PCR (qPCR) method. Cells were treated with 2.5 µM Bay-11-7082 to obtain the greatest loss of CD44 expression as determined in Western blotting (**Fig. 2**). In MDA-MB-231 cells, the mRNA level of NF-κB (48 and 72 hrs; **Fig. 3A**), CD44 (48 hrs; **Fig. 3B**), BCL-XL (48 and 72 hrs; **Fig. 3C**), and cMyc (72 hrs; **Fig. 3D**) decreased markedly after treatment. In SUM159 cells, decrease in the mRNA level of NF-κB (48 hrs; **Fig. 3E**), CD44 (48 and 72 hrs; **Fig. 3F**), and cMyc (48 hrs; **Fig. 3H**) was observed. A trend of significant decrease was seen after 48 hrs. However, after 72 hrs, the mRNA level of NF-κB and cMyc was increased to a level similar to the control DMSO treatment. No obvious difference was seen in BCL-XL mRNA after treatment.

The qPCR results correlated well with the results from both EMSA and Western blotting, suggesting that Bay-11-7082 inhibits NF-κB expression at both mRNA and protein level. Furthermore, NF-κB inhibition via Bay-11-7082 treatment represses the expression of CD44 and other NF-κB target genes, e.g., BCL-XL and cMyc.

### NF-κB inhibition induced CD44 repression decreases cell proliferation in breast cancer cells

To determine the effect of NF-κB inhibition induced CD44 repression on breast cancer cell properties, we first examined cell morphology (e.g., size and CD44 staining pattern) after Bay-11-7082 treatment to determine if the cells were healthy after treatment. No obvious changes in morphology and cell size (**Fig. S2**) and CD44 staining pattern (**Fig. S3**) were observed in breast cancer cells treated with Bay-11-7082.

**Figure 4:**
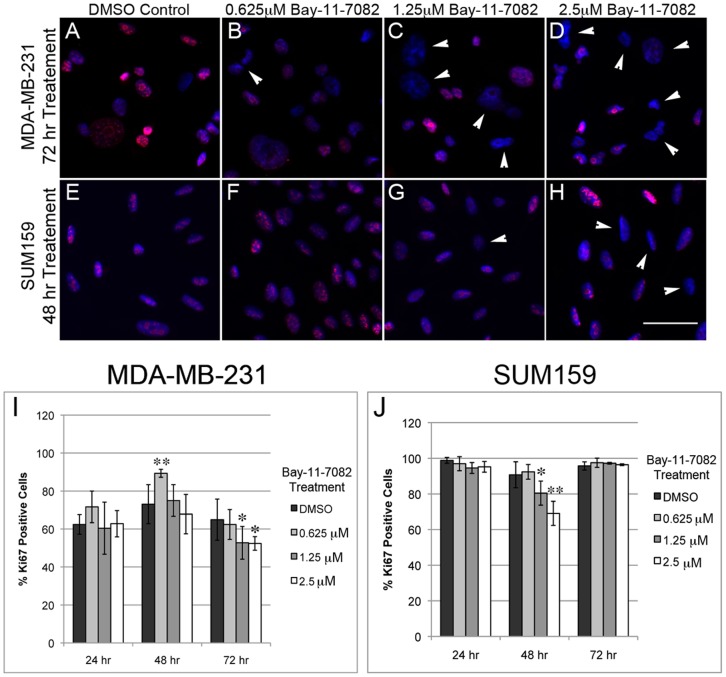
NF-κB inhibition and CD44 down-regulation result in decreased cell proliferation in breast cancer cells. Cell proliferation assays were performed using Ki67 staining. MDA-MB-231 (**A–D**) and SUM159 cells (**E–H**) were treated with either a DMSO control or Bay-11-7082 at different concentrations. Ki67 negative cells are indicated by arrowheads. Quantification showed decreased cell proliferation in MDA-MB-231 cells after 72 hrs treatment (**I**) and in SUM159 cells after 48 hrs treatment (**J**). (n = 3; * p≤0.05, ** p≤0.01). Scale bar = 50 µm.

Next, we performed a cell proliferation assay by immunostaining with Ki67, a nuclear protein associated with cell proliferation. A significant decrease in the percentage of Ki67 positive cells was observed with treatment of 1.25 µM and 2.5 µM Bay-11-7082 after 72 hrs in MDA-MB-231 cells (**Fig. 4A–D,I**) and after 48 hrs in SUM159 cells (**Fig. 4E–H,J**). An increase in the percentage of Ki67 positive cells was observed at 48 hrs treatment with 0.625 µM of Bay-11-7082 in MDA-MB-231 cells. This result may suggest that Bay-11-7082 stimulates cell proliferation at a low concentration. Interestingly, in SUM159 cells, the percentage of Ki67 positive cells was comparable to the DMSO control after 72 hrs treatment at all concentrations (**Fig**. **4J** **)**, suggesting that prolonged treatment has no lasting effect on cell proliferation in SUM159 cells, possibly due to development of drug resistance in this cell line.

### CD44 repression by inhibition of NF-κB binding to DNA decreases invasiveness and migration in breast cancer cells

**Figure 5:**
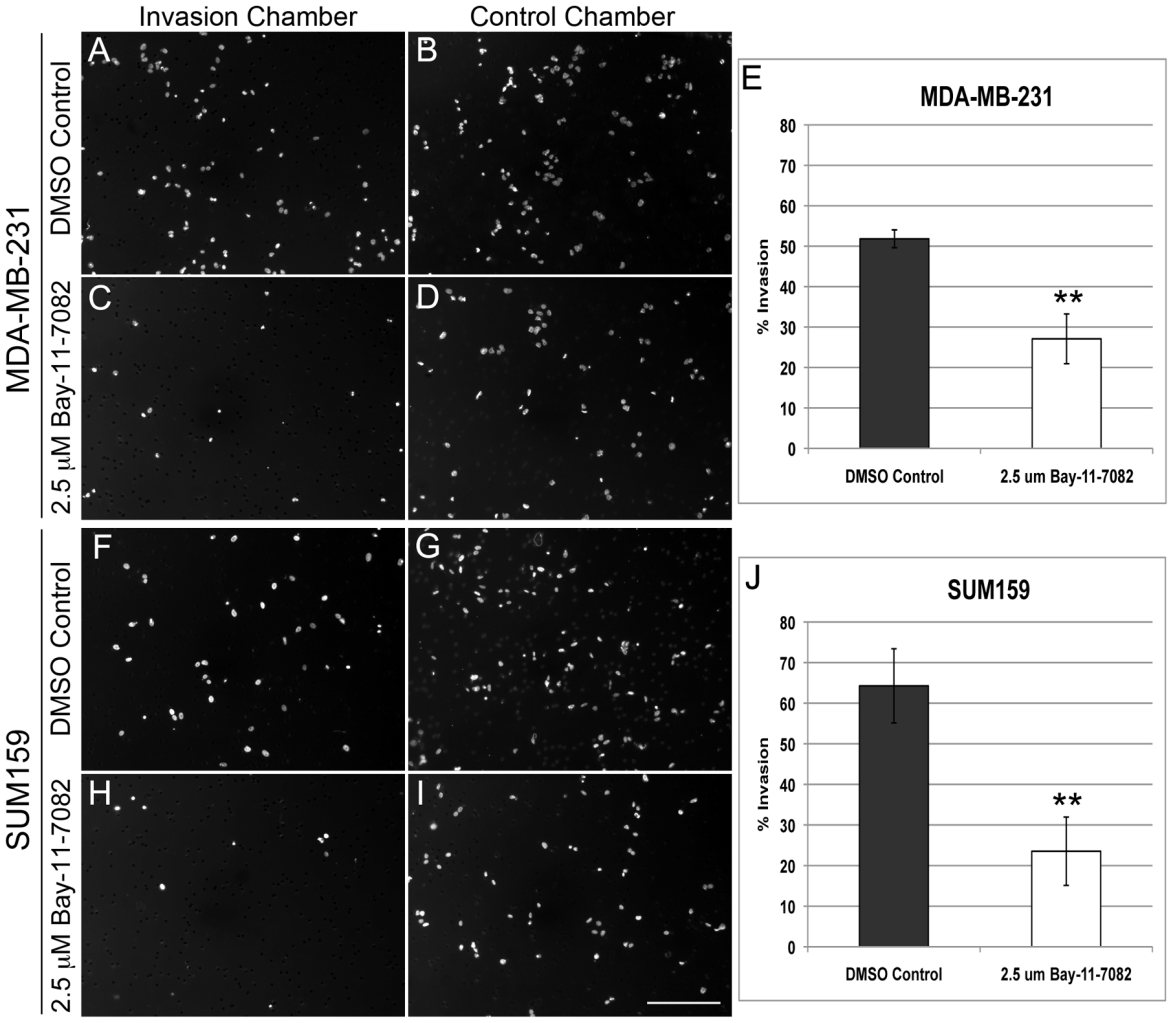
Bay-11-7082 treatment results in NF-κB inhibition and decreased invasiveness in breast cancer cells. An invasiveness assay was performed using matrigel with MDA-MB-231 (**A-D**) and SUM159 cells (**F–I**) after treatment with DMSO control or 2.5 µM Bay-11-7082. Quantification of the number of MDA-MB-231 (**E**) and SUM159 cells (**F**) penetrated the matrigel showed a decreased invasiveness in breast cancer cells after Bay-11-7082 treatment (n = 3; ** p≤0.01). Scale bar = 200 µm.

CD44 has previously been shown to play a role in migration and invasiveness of breast cancer cells [3], [26]. We, therefore, performed a matrigel invasion assay to determine the effect of CD44 down-regulation by NF-κB inhibition on the metastatic potential of breast cancer cells. Matrigel was used to block pores of a chamber membrane (invasion chamber) and, in-turn, prevent non-invading cells from migrating through the membrane. Cells with invasive properties will be able to migrate and penetrate through the matrigel and subsequently the membrane pores. As a control, cells were seeded into a control chamber containing no matrigel, just the porous membrane (control chamber). We found that the control DMSO treated MDA-MB-231 cells and SUM159 cells invaded both the matrigel and control chambers (**Fig. 5**). Quantification showed that 52% of the control DMSO treated MDA-MB-231 cells (**Fig. 5A–B,E**) and 64% of SUM159 cells (**Fig. 5F–G,J**) were able to invade the matrigel chamber (the number of cells in the control chamber was used as the base line). However, after cells were treated with 2.5 µM Bay-11-7082 for 72 hrs (with the greatest CD44 repression see **Figs 2** **–** **3**), only about 27% of MDA-MB-231 cells (**Fig. 5C–E**) and 24% of SUM159 cells (**Fig. 5H–J**) were able to invade the matrigel chamber. This significant decrease in number of cells invaded the matrigel chamber indicates that Bay-11-7082 treatment decreases the invasiveness of breast cancer cells. To assess the effect of Bay-11-7082 treatment on cancer cell migration, we quantified and compared the number of cells that penetrated the membrane pores in the control chamber (**Fig. 5B,D,G,I**). We observed a significant decrease in the percentage of cells that penetrated the membrane pores with Bay-11-7082 treatment as compared with DMSO control treatment (60% vs 40% in MDA-MB-231 cells (**Fig.S4A**) and 86% vs 14% in SUM159 cells (**Fig.S4B**)). Thus, NF-κB inhibition by Bay-11-7082 treatment decreases both invasiveness and migration in breast cancer cells.

## Discussion

In this study, we determined the effects of NF-κB inhibition on the expression of its target genes, e.g., CD44, BCL-XL, and cMyc, as well as proliferation, migration and invasiveness of breast cancer cells. We showed that the chemical compound Bay-11-7082 inhibits NF-κB activation by limiting NF-κB binding to DNA (**Fig. 1**). NF-κB inhibition causes a moderate decrease in CD44 expression at both the protein (**Fig. 2**) and mRNA (**Fig. 3**) level. In addition, repression of NF-κB target genes, e.g., CD44 and possibly other genes (e.g., BCL-XL, cMyc, and MMP9), decreased proliferation (**Fig. 4**) and invasiveness (**Fig. 5**) of breast cancer cells.

Previously studies have shown that CD44 expression in hepatoma and cervical cancer cells was regulated via NF-κB binding in the promoter of CD44 gene [27]. NF-κB was also identified as a regulator of CD44 expression in melanocytes, however, no NF-κB binding site in the CD44 promoter has been identified [28]. Thus, the molecular mechanism underlying NF-κB mediated CD44 regulation remains controversial. Our analysis of the human CD44 promoter confirmed that there were no NF-κB binding sites **(Table S1)**. We thus suggest that CD44 repression by NF-κB inhibition is via its binding to the CD44 *cis*-element CR1 [13]. Our previous studies using EMSA, ChIP, and site-directed mutagenesis and reporter assays have confirmed that NF-κB binds with CR1 and represses CD44 expression [13]. Our findings in this study, thus, established a direct correlation with NF-κB inhibition and CD44 repression in breast cancer cells, and provide new insight in the molecular mechanism of CD44 regulation.

As a therapeutic target, NF-κB is limited by its cross-talk with other pathways, poor drug specificity, and drug resistance [29]. Bay-11-7082 has been shown to prevent IKK (IκB kinase) from phosphorylating IκB (inhibitor of κB) thus preventing NF-κB from translocating to the nucleus to activate target genes [28]. Our study has found Bay-11-7082 was able to inhibit NF-κB binding to DNA in breast cancer cells at concentrations lower than previously reported [22], [30], [31]. Consistent with earlier studies performed on gastric cancer cells [32], [33], we found the use of Bay-11-7082 at higher concentrations was toxic to breast cancer cells and caused a significant amount of cell death that was time and dosage dependent. When NF-κB expression was silenced using retrovirus-mediated RNAi gene knockdown approach, we also observed a massive cell death (data not shown). These results allowed us to determine that a complete loss of NF-κB activation is not needed to obtain CD44 repression.

Equally important, our study showed a modest level of CD44 repression by NF-κB is sufficient to significantly reduce the cell proliferation and invasiveness of the triple negative breast cancer cells. This suggests that it is possible to achieve a therapeutic effect without a complete repression of CD44 and has an impact on future development of breast cancer treatment.

Despite a maximum of 30% decrease in CD44 expression at both the mRNA (qPCR in **Fig. 3**) and protein (Western blotting in **Fig. 2**) level, immunocytochemistry analysis of CD44 showed little difference in CD44 staining pattern (**Fig. S3**). Previous studies have shown CD44 expression can occur in sparsely dispersed patches or plaques [34]. These patterns of expression are important for CD44 cellular activities including cell-cell adhesion, migration and invasion. It is thus possible that such a small percentage decrease in CD44 expression on the surface of the cells is not detectable by immunocytochemistry. Further analysis will be needed to identify changes in expression in these patches and plaques [5], [35].

NF-κB-p65 phosphorylation has been implicated in the up-regulation of TICs in breast cancer. Following NF-κB inhibition, it was shown that the number of CD44 high expressing breast TICs diminished [36]. Up-regulation of CD44 has been shown to increase proliferation and invasiveness of cancer cells [3], [36], [37]. TICs, in particular, have been implicated in cancer progression and tumor cell proliferation [32], [38], [39].

Interestingly, cell proliferation was not affected in SUM159 cells following 72 hrs Bay-11-7082 treatment (**Fig. 4D**). Similarly, we found NF-κB binding as well as CD44 protein and RNA levels returned to its base level following 72 hrs of treatment in SUM159 cells (**Figs. 1** **–** **3**). This may be due to drug-resistance in SUM159 cells as they are triple negative breast cancer cells and known to develop chemotherapy resistance [40], [41]. Multiple drug resistance in SUM159 cells is one of the major causes resulting in increased severity of breast cancer [1], [42]. Therefore, it is possible that SUM159 cells develop resistance to Bay-11-7082 treatment after prolonged exposure.

Cancer cells with up-regulated CD44 expression are responsible for metastasis in breast cancer [26], [35], [43]. High expression of CD44 coupled with low expression of CD24 has been shown to correlate with an invasive phenotype [35]. Our observation that CD44 repression results in decreased invasiveness (**Fig. 5**) and migration (**Fig. S4**) in breast cancer cells is consistent with the notion that CD44 expression is one of the key determinants of the migration and invasiveness of cancer cells. NF-κB has also been shown to decrease proliferation and invasiveness via its regulation of matrix metalloproteinase 9 (MMP9) [44]–[47]. MMP9, along with numerous inflammation-related cytokines and chemokines, could have affected cell behavior and contributed to the observed cellular phenotype. CD44 and MMP9 have previously been shown to form a complex and together promote invasiveness in cancer cells [37], [48]–[50]. Thus, repression of CD44 and MMP9 by the inhibition of NF-κB could be responsible for the decreased invasiveness seen in breast cancer cells.

It is also important to note that the loss of only 30% of CD44 expression due to NF-κB inhibition is not uncommon when studying regulation at the enhancer level. A recent study of a PTF1A enhancer in the pancreas found that point mutations had the ability to decrease enhancer activity in half or completely, depending on the specific mutation [51]. Therefore, we suggest that CD44 is regulated by the interaction of NF-κB with the cis-element CD44CR1, however, we do not rule out other proteins or regulator regions responsible for the up-regulation of CD44 in cancer and TICs.

Together, our data suggest that targeting NF-κB activation reduces the expression of its target genes (e.g., CD44, BCL-XL,and cMyc) and subsequently affects proliferation and invasiveness of triple negative breast cancer cells. Future studies, such as xenograft models, will be needed to confirm these findings *in vivo*. Furthermore, analysis of other transcription factors that bind to CD44CR1, e.g., AP-1 [13], may prove to have a synergetic effect on CD44 expression and cellular activities. Thus, our findings provide potential therapeutic targets in the fight against breast cancer.

## Supporting Information

Figure S1 **High concentration of Bay-11-7082 causes dramatic cell death.** Significant cell death occurs in MDA-MB-231 (**A**) and SUM159 cells (**B**) when treated with 5.0 µM and 10.0 µM Bay-11-7082 after 24 hrs, 48 hrs, and 72 hrs of treatment. 100% cell death was seen with 10 µM treatment. Δ represents complete cell death at 10.0 µM treatment (n = 3; * p≤0.05, ** p≤0.01).
(TIF)

Figure S2 **Bay-11-7082 treatment does not affect cell size.** MDA-MB-231 (**A**) and SUM159 cells (**B**) treated with different concentrations of Bay-11-7082 showed no significant changes in cell size following 24 hrs, 48 hrs, or 72 hrs of treatment at any concentration.
(TIF)

Figure S3 **Immunocytochemistry does not reveal significant changes in cell surface expression of CD44 in breast cancer cells.** Immunostaining of breast cancer cells with CD44 antibody following treatment with Bay-11-7082. MDA-MB-231 (**A-L**) and SUM159 cells (**M-X**) showed no obvious changes in CD44 expression after Bay-11-7082 treatment for 24 hrs, 48 hrs, and 72 hrs. Scale bar = 50 µm.
(TIF)

Figure S4 **Bay-11-7082 treatment decreases cell migration in breast cancer cells.** Migration assays were performed using control chamber with MDA-MB-231 (see **Fig. 5B, D**) and SUM159 cells (see **Fig. 5G,I**) after treatment with either a DMSO control or 2.5 µM Bay-11-7082. Quantification showed a significant decrease in the percentage of MDA-MB-231 (**A**) and SUM159 cells (**B**) penetrated the membrane pores in the control chamber (n = 3; ** p≤0.01).
(TIF)

Table S1 **Transcriptioin factor binding sites on CR1 of CR44 locus as predicted using Genomatix.**
(XLS)
