# Supplementary figures and images for: NF-κB Affects Proliferation and Invasiveness of Breast Cancer Cells by Regulating CD44 Expression
Source: PLoS One. 2014 Sep 3;9(9):e106966. doi: 10.1371/journal.pone.0106966 (PMC4153718; doi:10.1371/journal.pone.0106966)

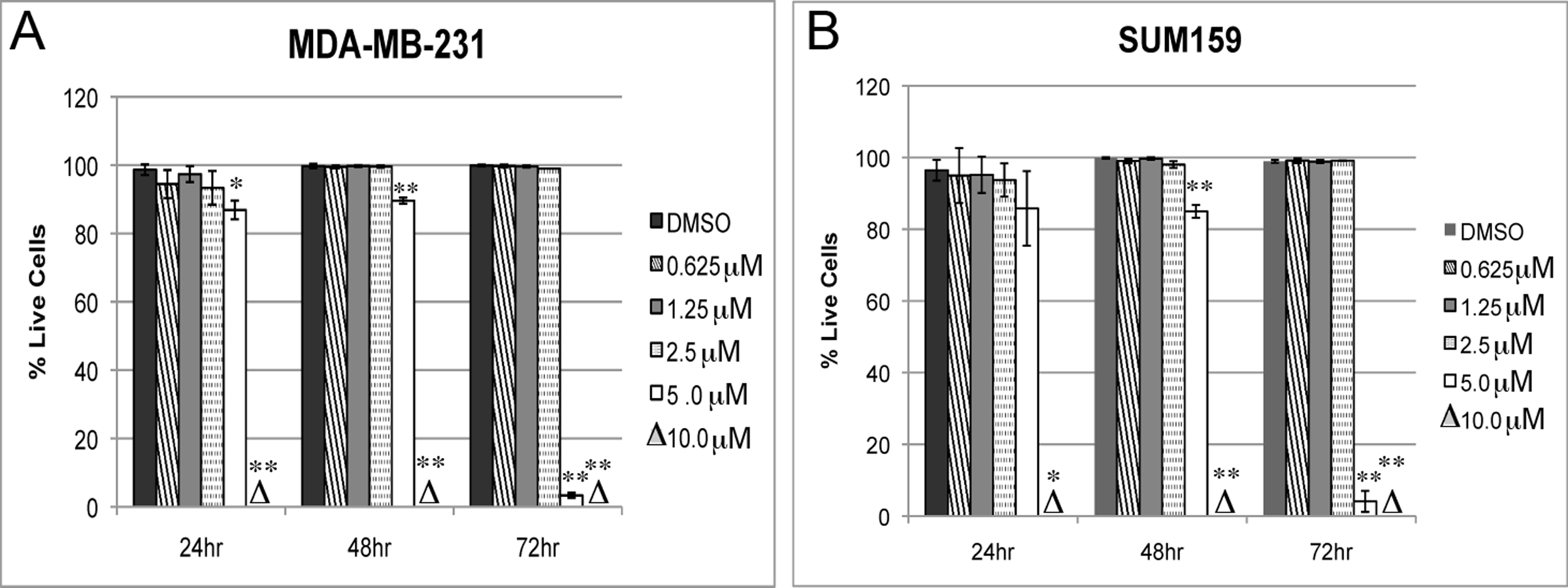

Supplement: Figure S1 — High concentration of Bay-11-7082 causes dramatic cell death. Significant cell death occurs in MDA-MB-231 (A) and SUM159 cells (B) when treated with 5.0 µM and 10.0 µM Bay-11-7082 after 24 hrs, 48 hrs, and 72 hrs of treatment. 100% cell death was seen with 10 µM treatment. Δ represents complete cell death at 10.0 µM treatment (n = 3; * p≤0.05, ** p≤0.01). (TIF) [file pone.0106966.s001.tif]

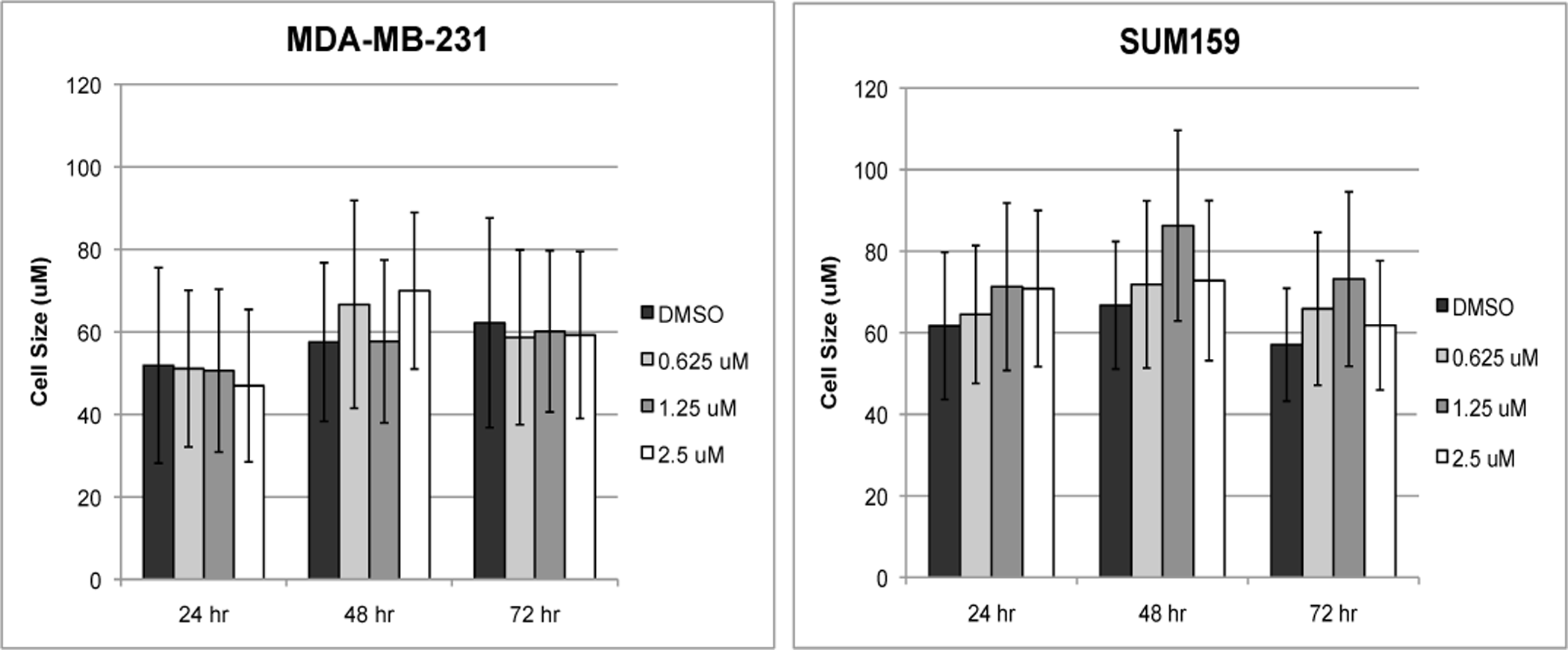

Supplement: Figure S2 — Bay-11-7082 treatment does not affect cell size. MDA-MB-231 (A) and SUM159 cells (B) treated with different concentrations of Bay-11-7082 showed no significant changes in cell size following 24 hrs, 48 hrs, or 72 hrs of treatment at any concentration. (TIF) [file pone.0106966.s002.tif]

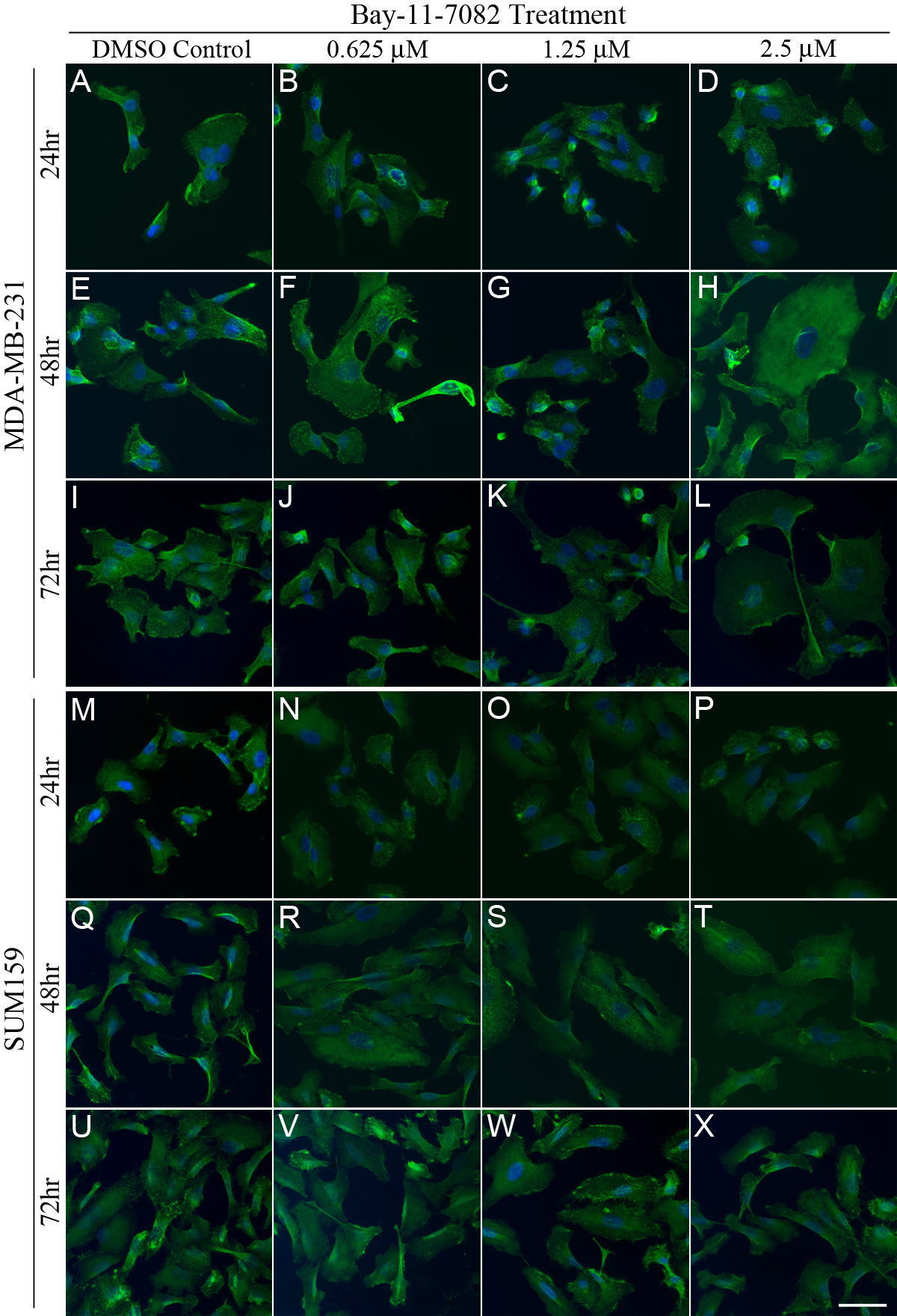

Supplement: Figure S3 — Immunocytochemistry does not reveal significant changes in cell surface expression of CD44 in breast cancer cells. Immunostaining of breast cancer cells with CD44 antibody following treatment with Bay-11-7082. MDA-MB-231 (A-L) and SUM159 cells (M-X) showed no obvious changes in CD44 expression after Bay-11-7082 treatment for 24 hrs, 48 hrs, and 72 hrs. Scale bar = 50 µm. (TIF) [file pone.0106966.s003.tif]

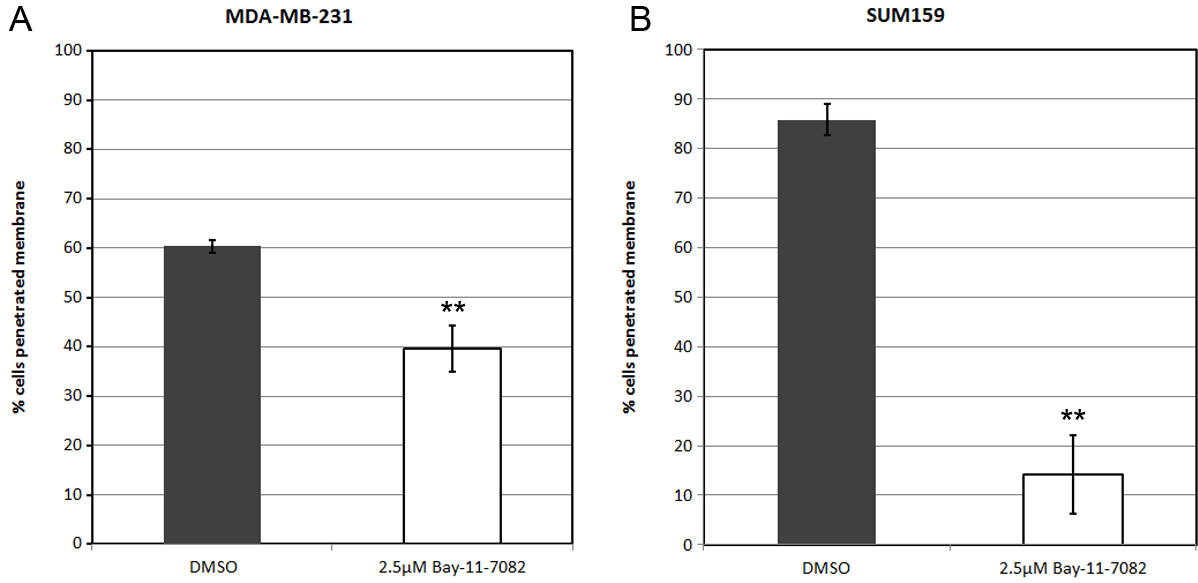

Supplement: Figure S4 — Bay-11-7082 treatment decreases cell migration in breast cancer cells. Migration assays were performed using control chamber with MDA-MB-231 (see Fig. 5B, D ) and SUM159 cells (see Fig. 5G,I ) after treatment with either a DMSO control or 2.5 µM Bay-11-7082. Quantification showed a significant decrease in the percentage of MDA-MB-231 (A) and SUM159 cells (B) penetrated the membrane pores in the control chamber (n = 3; ** p≤0.01). (TIF) [file pone.0106966.s004.tif]
